# Commentary: Astroglial CB_1_ Receptors Determine Synaptic D-Serine Availability to Enable Recognition Memory

**DOI:** 10.3389/fphar.2018.00988

**Published:** 2018-08-31

**Authors:** Yan-Chen Guo, Ti-Fei Yuan

**Affiliations:** ^1^School of Psychology, Nanjing Normal University, Nanjing, China; ^2^Shanghai Key Laboratory of Psychotic Disorders, Shanghai Mental Health Center, Shanghai Jiao Tong University School of Medicine, Shanghai, China; ^3^Co-innovation Center of Neuroregeneration, Nantong University, Nantong, Jiangsu, China

**Keywords:** CB_1_ receptors, D-serine levels, memory, LTP, astrocytes

Accumulating evidence demonstrated that glial cells, especially the astrocytes participated in neuronal communication and regulation of synaptic plasticity (Panatier et al., 2008; Henneberger et al., 2010). In contrast to the presynaptic inhibitory roles, the type-1 cannabinoid receptors (CB_1_) expressed on astrocytes play a crucial role in glial-neuronal interactions and potentiate synaptic transmission (Navarrete and Araque, 2010). In addition, CB_1_ receptors activation on astrocytes leads to release of D-serine, which modulates synaptic NMDARs and regulates LTP in hippocampus (Papouin et al., 2012). On the other hand, another study showed that activation of astroglial CB_1_ receptors resulted in LTD induction at CA3-CA1 synapses and impaired spatial working memory (Han et al., 2012). However, it remains unknown whether astroglial CB_1_ receptors regulate D-serine/NMDA receptor signaling during long-term memory processes.

In the recent study, Robin et al. found that CB_1_ receptors in hippocampal astrocytes facilitated long-term novel object recognition (NOR) memory consolidation, by D-serine/NMDA receptor signaling (Robin et al., 2018). The authors firstly found that GFAP-CB_1_-KO mice displayed LTP induction and long-term NOR memory deficits as compared to their counterparts, similar to animals treated with NMDARs antagonist. WIN55, 212-2(CB_1_ receptor agonist) induced-increase of Ca^2+^ levels in hippocampal astrocyte was lost in GFAP-CB_1_-KO mice. In preparations of brain slice filed recording with D-serine perfusion, GFAP-CB_1_-KO mice had twice more increased synaptic response than GFAP-CB_1_-WT mice, suggesting for tonic suppression of synaptic functions due to D-serine deficiency; in addition, the reduced LTP in hippocampal slice from GFAP-CB_1_-KO mice could be rescued by exogenous application of D-serine.

The researchers then employed multiple approaches to increase D-serine levels *in vivo* and found that the memory deficits of GFAP-CB_1_-KO mice could be rescued by increase of endogenous or exogenous D-serine levels. Moreover, mice lacking CB_1_ gene specifically in hippocampal astrocytes demonstrated similar memory deficits that could be reversed by systemic injection of D-serine as the GFAP-CB_1_-KO mice. This excluded the possibility that alterations of D-serine levels in the hippocampus were resulted from deletion of CB_1_ receptors in other brain regions.

Previous study blocked hippocampal LTP induction by clamping internal Ca^2+^ in individual CA1 astrocytes, which could be reversed by exogenous D-serine, whereas disruption of D-serine exocytosis and depletion of the stored D-serine in an individual astrocyte blocked local LTP (Henneberger et al., 2010). Robin et al. further explored the mechanism of recognition memory involving the astroglial CB_1_ receptors. Notably, the poor performance of GFAP-CB_1_-KO mice in recognition memory could be rescued via the increase of the D-serine levels. In addition, GFAP-CB_1_-KO mice had twice more increased synaptic response to application of the D-serine than GFAP-CB_1_-WT mice; these data suggested that astroglial CB_1_ receptors play a crucial role in maintaining appropriate concentrations of extracellular D-serine to keep NMDAR co-agonist sites unsaturated in baseline conditions (Henneberger et al., 2010; Robin et al., 2018).

The present study demonstrated the role of CB_1_ receptors in astrocytes and one of gliotransmitters D-serine in recognition memory. In fact, the acquisition and consolidation of memory also recruit other neuron–glia signaling pathways. For instance, Vignoli et al. found that astrocytes were engaged in BDNF recycling; mice deficient in BDNF recycling displayed NOR memory deficits (Vignoli et al., 2016). Moreover, in addition to D-serine, astrocytes in hippocampus can release glutamate and ATP, which might modulate LTP or LTD via different mechanisms (Navarrete et al., 2012; Schmitt et al., 2012).

There were still some limitations for the present study. For instance, the effects of D-serine on presynaptic NMDARs and postsynaptic NMDARs remain unknown. Presynaptic NMDARs are also required for LTP at cortical projections to striatum (Humeau et al., 2003; Park et al., 2014), and may contribute to the enhancement of glutamate release at CA3 to CA1 synapses after LTP induction (Mcguinness et al., 2010). Astrocytes exchange information with both presynaptic terminals and post-synaptic receptors, and this existence of bidirectional communication established the concept of “tripartite synapse” (Figure 1) (Araque et al., 1999; Perea et al., 2009; Oliveira da Cruz et al., 2015). In tripartite synapse, astrocytes can release multiple gliotransmitters and even a single gliotransmitter can activate different target (Araque et al., 2014). For instance, astrocytes can release D-serine acted as a co-agonist of the postsynaptic NMDARs necessary for the induction of hippocampal LTP (Henneberger et al., 2010), and D-serine can also act as a co-agonist of the presynaptic NMDARs, which is necessary for the induction of hippocampal t-LTD (Andrade-Talavera et al., 2016). Last but not least, there is no direct evidence showing how the increases of calcium level in astrocytes control the release of D-serine.

**Figure 1:**
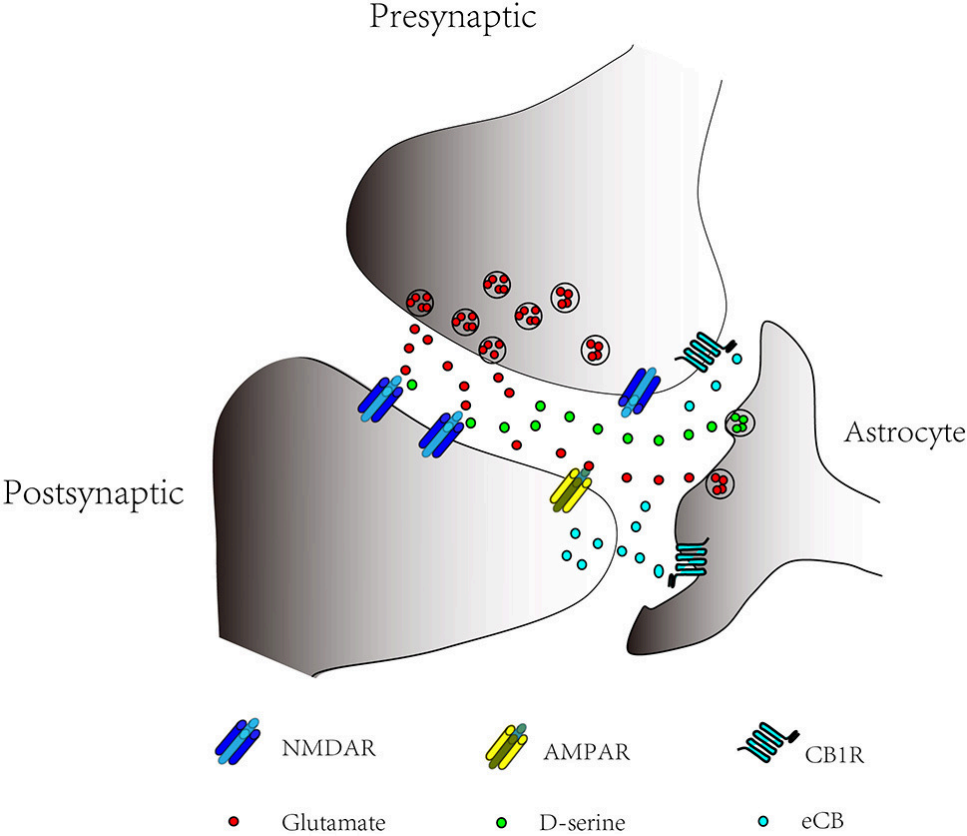
Bidirectional Communication in Tripartite Synapse. Astrocytes exchange information with the pre-and postsynaptic neurons. Endocannabinoids (eCBs) can bind to astrocytic CB1 receptors (CB_1_Rs) and presynaptic CB_1_Rs. Activation of astrocytic CB_1_Rs results in increase of Ca^2+^ levels in astrocytes; astrocytic release of glutamate or D-serine can bind to postsynaptic and presynaptic NMDARs to modulate LTP and LTD.

To sum, neuron-glia interaction are important regulators in memory network, especially through astrocytic CB_1_R-mediated D-serine release and the modulation on synaptic functions.

## Author contributions

Y-CG completed the first version of the paper and T-FY presented suggestions to revise the paper. The workload of the two authors was equal.

### Conflict of interest statement

The authors declare that the research was conducted in the absence of any commercial or financial relationships that could be construed as a potential conflict of interest.
